# Multipathway pharmacological interventions targeting liver-derived immune resistance in colorectal cancer liver metastasis

**DOI:** 10.3389/fimmu.2026.1978050

**Published:** 2026-09-25

**Authors:** Miaomiao Chen, Shanqing Qin, Yifeng Zhan, Qi Wang

**Affiliations:** Department of Pediatric Intensive Care Medicine, Hainan Women and Children’s Medical Center, Hainan Medical University School of Pediatrics, Haikou, China

**Keywords:** colorectal cancer, CRLM, immune resistance, immunotherapy, liver metastasis

## Abstract

Immune checkpoint inhibitors provide durable benefit in mismatch repair-deficient colorectal cancer, but most microsatellite-stable tumors remain resistant, particularly in the presence of liver metastases. Immune resistance in colorectal cancer liver metastasis reflects both baseline MSS-CRC mechanisms, including low neoantigenicity, limited T-cell priming and tumor-intrinsic immune exclusion, and liver-specific or liver-amplified mechanisms, including tolerogenic antigen presentation by hepatic resident cells, macrophage-mediated loss of tumor-reactive T cells, and hepatic stromal, vascular and metabolic remodeling. Across early-phase and retrospective studies, responses to checkpoint-based combinations have been observed more often in patients without active liver metastases, but these exploratory subgroup findings do not yet establish independent predictive value or clinical causality. Antiangiogenic agents, multikinase inhibitors, myeloid- and stromal-targeted therapies, innate immune activation, and liver-directed interventions may partially overcome these barriers, but single-pathway strategies have shown limited efficacy. This Mini Review summarizes the mechanisms and clinical evidence of liver-mediated immune resistance and proposes organ-informed, mechanism-matched therapeutic strategies for colorectal cancer liver metastasis.

## 1 Introduction

Colorectal cancer (CRC) remains a major cause of cancer-related mortality, largely because of metastatic disease (1). The liver is the predominant site of distant spread, and many patients develop colorectal cancer liver metastases (CRLM) during the course of their disease (2). Although resection or ablation can provide prolonged survival in selected patients, most CRLMs are unresectable at diagnosis or eventually recur, highlighting the need for more effective systemic strategies (3, 4). Immune checkpoint inhibitors (ICIs) have transformed the treatment of several solid tumors, but their efficacy in CRC is strongly dependent on molecular subtype. Durable responses are observed mainly in mismatch repair-deficient or microsatellite instability-high (dMMR/MSI-H) tumors, whereas the much larger mismatch repair-proficient or microsatellite-stable (pMMR/MSS) population generally derives little benefit from checkpoint blockade alone (5–7). This disparity has been attributed to lower neoantigen burden, limited T-cell infiltration and a less inflamed tumor microenvironment in MSS disease (8, 9).

However, molecular subtype alone does not fully explain the heterogeneous response of metastatic CRC to immunotherapy. Increasing evidence indicates that the anatomical site of metastasis can independently shape antitumor immunity. In particular, liver involvement has repeatedly been associated with reduced response rates and poorer outcomes in patients receiving ICIs or ICI-based combinations (10). Clinically, liver involvement is consistently associated with poorer outcomes during ICI treatment, but available analyses do not reliably separate a treatment-specific predictive effect from adverse prognostic factors such as tumor burden, performance status and disease biology. Experimental studies support a causal contribution of hepatic lesions in selected models, whereas the clinical evidence remains predominantly associative (11).

The liver is physiologically adapted to maintain immune tolerance because it is continuously exposed to dietary antigens, microbial products and metabolites delivered through the portal circulation (12–15). These hepatic-resident features are more organ-specific than low neoantigen burden, tumor-intrinsic immune exclusion, T-cell exhaustion, VEGF signaling or metabolic competition, which also occur in MSS CRC outside the liver. In CRLM, however, these shared programs may be amplified through interactions with Kupffer cells, liver sinusoidal endothelial cells, hepatic stellate cells and recruited myeloid populations. Experimental studies further show that the same CRC model can display markedly different immune infiltration and ICI sensitivity when established subcutaneously, orthotopically or within the liver, emphasizing the importance of organ context (9). Liver metastases may also suppress immunity beyond the hepatic lesions by reducing systemic tumor-reactive T-cell activity and impairing responses at extrahepatic sites (16, 17).

These observations help explain why intensified checkpoint blockade and multikinase inhibitor combinations have produced activity in selected patients but have frequently failed to generate consistent benefit in those with active liver metastases (18). Successful treatment may therefore require simultaneous correction of several organ-specific barriers rather than generalized enhancement of T-cell activity alone. In this Mini Review, we summarize the liver-specific and liver-amplified mechanisms associated with local and systemic immune resistance in CRLM, evaluate clinical evidence supporting liver metastasis as a modifier of immunotherapy response, and discuss pharmacological and liver-directed strategies designed to overcome this resistance. We further consider how liver-specific biomarkers and clinically representative models could support a shift toward organ-informed, mechanism-matched immunotherapy for metastatic CRC. The distinct contribution of this review is a three-axis state–mechanism–evidence framework that links hepatic disease status, the dominant cancer–immunity-cycle bottleneck and the maturity of supporting evidence to the selection of mechanism-matched combinations. Unlike broader liver-cancer reviews organized mainly by regimen class, this framework restricts efficacy claims to CRLM-specific data and labels hepatocellular carcinoma (HCC)-derived concepts as contextual rather than directly transferable.

## 2 Liver-specific and liver-amplified mechanisms of immune resistance

The limited responsiveness of CRLMs to immunotherapy reflects multiple interacting barriers rather than a single inhibitory pathway. Metastatic CRC cells enter an organ physiologically adapted to restrain excessive immune activation and subsequently reshape this baseline tolerance into a tumor-supportive ecosystem. Hepatic resident cells, recruited myeloid populations, dysfunctional lymphocytes, activated stroma, abnormal vasculature and metabolic stress jointly impair several stages of the cancer–immunity cycle, helping explain why intensified PD-1 or CTLA-4 blockade alone is often insufficient in established CRLM. For clarity, this section distinguishes baseline MSS-CRC resistance mechanisms from hepatic-resident mechanisms that are anatomically specific to the liver and from shared immunosuppressive programs that are not unique to the liver but may be amplified after hepatic colonization.

### 2.1 Tolerogenic hepatic immunity and myeloid remodeling

The liver is continuously exposed to dietary antigens, microbial products and metabolites delivered through the portal circulation. To prevent unnecessary inflammation, antigen presentation in the liver often occurs under regulatory conditions. Liver sinusoidal endothelial cells can capture circulating antigens but provide limited co-stimulation, favoring T-cell hyporesponsiveness or deletion (19, 20). Kupffer cells produce immunoregulatory mediators such as IL-10 and TGF-β, while hepatic stellate cells contribute to immune regulation and tissue repair. CRLM therefore develops within an organ where the threshold for productive immune activation is already relatively high (21).

Metastatic colonization amplifies this tolerance by reshaping the hepatic myeloid compartment. Although Kupffer cells can initially eliminate incoming tumor cells, persistent tumor-derived signals recruit circulating monocytes and redirect resident macrophages toward metastasis-supporting states. These cells promote tumor growth, angiogenesis and extracellular-matrix remodeling while suppressing adaptive immunity through IL-10, TGF-β, arginase activity, reactive oxygen species and checkpoint-ligand expression. Neutrophils and myeloid-derived suppressor cells further inhibit T-cell proliferation, alter nutrient availability and support vascular remodeling (22).

A particularly important mechanism is the macrophage-mediated removal of activated tumor-reactive lymphocytes. Liver metastases can recruit circulating antigen-specific CD8^+^ T cells, which subsequently undergo Fas-dependent apoptosis after interacting with FasL-expressing monocyte-derived macrophages. This process can reduce both local lymphocyte activity and the systemic pool of tumor-reactive T cells, thereby weakening immune control at extrahepatic sites (23). Defective dendritic-cell function creates an additional upstream barrier. In pMMR CRC liver metastasis models, low intratumoral dendritic-cell abundance limits antigen presentation and T-cell priming, reducing the efficacy of checkpoint blockade even when tumor antigens are present. Because PD-1 inhibition mainly reinvigorates pre-existing antitumor T cells, it is unlikely to be sufficient when antigen uptake, dendritic-cell activation and T-cell priming remain impaired (24).

Single-cell studies further indicate that macrophages, dendritic cells and neutrophils in CRLM occupy diverse functional states (25). This heterogeneity argues against broad myeloid depletion, which could also eliminate beneficial phagocytic or antigen-presenting populations. Selective blockade of suppressive recruitment pathways or functional reprogramming of metastasis-supporting myeloid states may therefore be more appropriate.

### 2.2 T-cell exclusion, dysfunction and systemic suppression

T-cell exclusion and exhaustion also occur in MSS CRC outside the liver and should not, by themselves, be considered liver-specific; in CRLM, their liver-amplified component arises when hepatic tolerance and myeloid remodeling converge on several forms of T-cell failure. Some CRLMs contain few intratumoral effector T cells because dendritic-cell scarcity, abnormal vasculature and suppressive chemokine networks limit priming and recruitment. In other lesions, T cells accumulate at the invasive margin or within stromal regions but remain spatially separated from malignant cells. Even when lymphocytes enter the tumor, chronic antigen exposure, insufficient co-stimulation and inhibitory cytokines can induce dysfunctional states characterized by reduced cytokine production and cytotoxicity. These phenotypes represent distinct therapeutic bottlenecks. Low T-cell abundance reflects defective immune generation or recruitment; spatial exclusion indicates stromal or vascular restriction; and exhaustion reflects impaired function within the lesion. Treating all three states simply by adding checkpoint inhibitors is therefore unlikely to produce equivalent benefit.

Experimental comparisons between implantation sites reinforce the importance of organ context. The same CRC cell line can show lower T-cell and dendritic-cell infiltration and greater checkpoint resistance when established orthotopically or in the liver than when grown subcutaneously (24). Thus, tumor genotype alone does not determine immune responsiveness, and highly immunogenic subcutaneous models may overestimate therapeutic efficacy. CRLM may also suppress immunity beyond the liver. Macrophage-mediated deletion of circulating tumor-reactive CD8^+^ T cells can reduce immune control of simultaneously growing extrahepatic lesions, suggesting that the hepatic metastasis functions as an active systemic immunoregulatory compartment. Regulatory T cells and suppressive myeloid signals may further reinforce this effect (26).

Chronically stimulated T cells in CRLM can co-express PD-1, TIM-3, LAG-3 and TIGIT (27). However, high checkpoint expression does not necessarily predict sensitivity to combined blockade. If the dominant defect is loss of antigen-presenting cells, physical exclusion or deletion of tumor-reactive clones, additional checkpoint inhibition may act on only a limited residual T-cell population. Tumor-intrinsic programs can further reinforce this state; for example, WNT11 signaling has been associated with T-cell exclusion and anti-PD-1 resistance in CRLM models (28). These observations emphasize that hepatic resistance arises from reciprocal interactions between metastatic tumor cells and the host organ.

### 2.3 Liver-amplified stromal, vascular and metabolic barriers

Stromal exclusion, abnormal vasculature, hypoxia and metabolic competition occur across solid tumors and are not intrinsically liver-specific; in CRLM, however, metastatic growth can reorganize the hepatic stromal and vascular architecture. Activated hepatic stellate cells and cancer-associated fibroblasts produce collagen, fibronectin and other extracellular-matrix components, generating a fibrotic interface that restricts immune-cell movement. TGF-β promotes fibroblast activation, matrix deposition and suppressive myeloid differentiation, while CXCL12–CXCR4 signaling can retain lymphocytes within stromal compartments rather than allowing direct contact with tumor cells (29, 30). The spatial location of immune cells may therefore be as important as their overall abundance.

Fibroblasts are nevertheless functionally heterogeneous. Single-cell and spatial analyses reveal both suppressive fibrotic states and immune-supportive stromal niches (31, 32). CCL19-producing fibroblasts, for example, can recruit lymphocytes and promote tertiary lymphoid structure formation, supporting local antitumor immunity (33). These findings argue against indiscriminate fibroblast depletion and instead support selective targeting of stromal programs directly responsible for immune exclusion. Abnormal tumor vasculature adds another layer of resistance. VEGF-driven vessels are structurally disorganized and may express insufficient adhesion molecules for efficient lymphocyte trafficking. Poor perfusion promotes hypoxia, which supports suppressive macrophages, regulatory T cells and angiogenic signaling (34). Hypoxic regions also accumulate lactate and extracellular adenosine, impairing effector T-cell and natural killer-cell activity (35, 36). Tumor cells can restrict glucose availability, while arginase-producing myeloid cells deplete extracellular arginine and suppress antigen-specific T-cell responses (37).

These pathways form a self-reinforcing circuit rather than parallel barriers. Tolerogenic liver sinusoidal endothelial cells and Kupffer cells, together with dendritic-cell paucity, limit productive priming and reduce the supply of tumor-reactive T cells. VEGF-driven vascular dysfunction and CAF-derived matrix then restrict lymphocyte entry and worsen hypoxia, while hypoxia, lactate and adenosine favor the recruitment or reprogramming of suppressive macrophages, neutrophils and myeloid-derived suppressor cells. These myeloid populations further release VEGF, TGF-β, IL-10 and arginase, reinforcing fibroblast activation, abnormal perfusion, defective antigen presentation and T-cell loss or dysfunction. Reduced cytotoxicity and IFN-γ signaling in turn weaken antigen spreading and vascular activation, closing the feedback loop. This model explains why blockade of a single pathway often leaves compensatory arms intact and why the dominant bottleneck may differ among lesions.

Overall, the CRLM resistance phenotype reflects the superimposition of baseline MSS-CRC immune coldness on liver-specific or liver-amplified failures in three linked processes: generation and maintenance of tumor-reactive immunity, survival and function of effector T cells, and access to spatially and metabolically protected hepatic metastatic niches. Their relative contribution is likely to vary between patients and between lesions within the same patient. Effective treatment will therefore require moving beyond a simple “hot” versus “cold” classification toward identifying whether resistance is dominated by defective priming, myeloid-mediated deletion, stromal exclusion or vascular–metabolic dysfunction. These interconnected mechanisms and their corresponding mechanism-matched therapeutic strategies are summarized in **Figure 1**.

**Figure 1:**
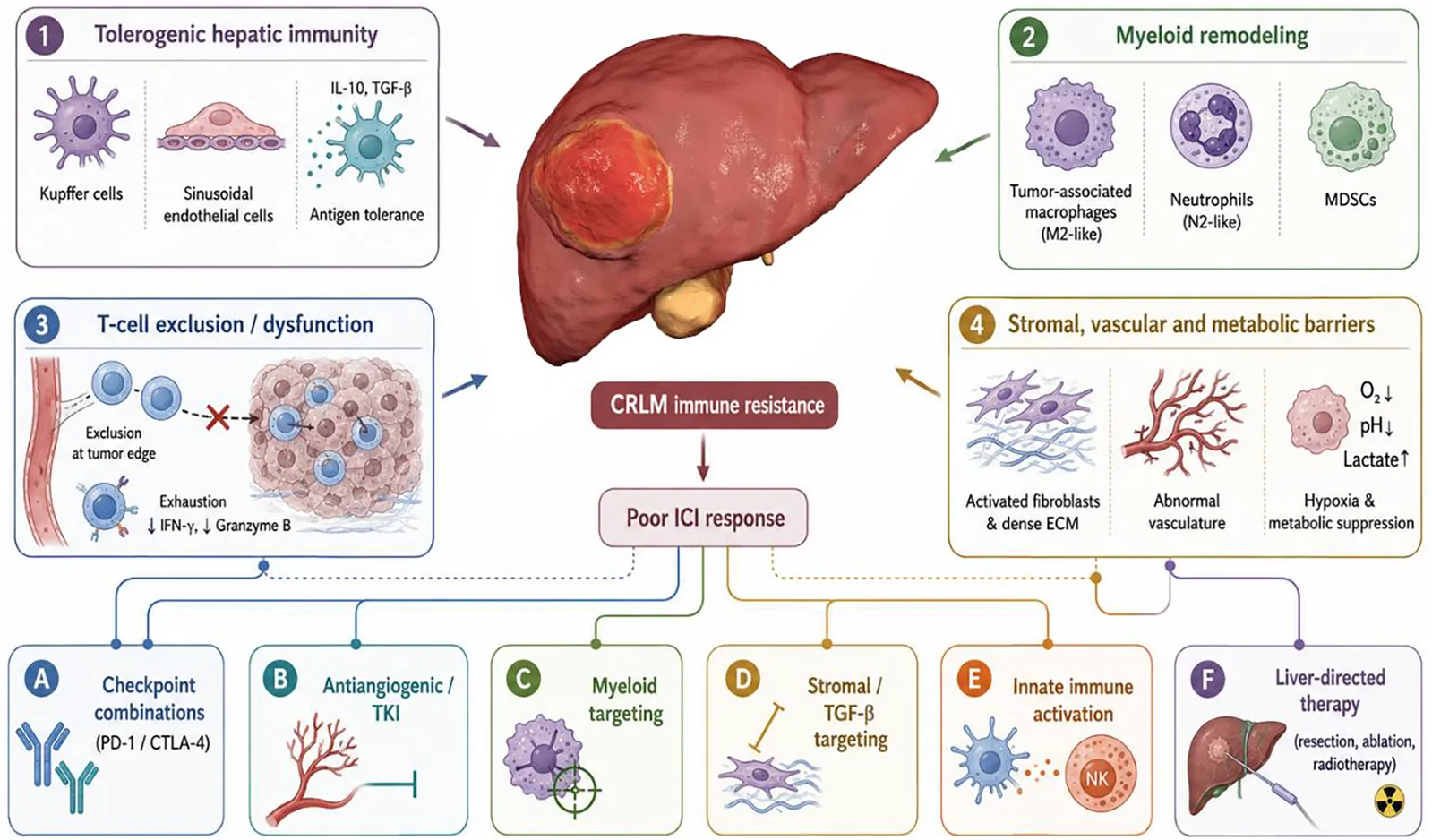
Liver-mediated immune resistance and mechanism-matched therapeutic strategies in colorectal cancer liver metastasis. Four interconnected mechanisms establish the immunosuppressive hepatic ecosystem: ① tolerogenic hepatic immunity mediated by Kupffer cells, liver sinusoidal endothelial cells and IL-10/TGF-β-associated antigen tolerance; ② myeloid remodeling involving tumor-associated macrophages, N2-like neutrophils and myeloid-derived suppressor cells; ③ T-cell exclusion and dysfunction, including retention at the tumor margin, exhaustion and reduced IFN-γ and granzyme B production; and ④ stromal, vascular and metabolic barriers characterized by activated fibroblasts, dense extracellular matrix, abnormal vasculature, hypoxia, acidosis and lactate accumulation. These processes promote CRLM immune resistance and poor ICI response. Corresponding interventions include checkpoint combinations **(A)**, antiangiogenic agents or TKIs **(B)**, myeloid targeting **(C)**, stromal or TGF-β targeting **(D)**, innate immune activation **(E)** and liver-directed therapy **(F)**.

## 3 Clinical evidence and pharmacological interception

Clinical observations are consistent with, but do not by themselves prove, liver-mediated immune resistance. Across multiple studies of microsatellite-stable or mismatch repair-proficient metastatic colorectal cancer (MSS/pMMR mCRC), responses to immune checkpoint inhibitor (ICI)-based combinations have occurred disproportionately in patients without active liver metastases. However, liver involvement is also associated with greater tumor burden, adverse disease biology and extensive prior treatment, making it difficult to distinguish a predictive effect from a generally unfavorable prognosis. The available evidence should therefore be interpreted as a consistent organ-specific clinical signal rather than definitive proof that liver metastasis is an independently validated biomarker. Nevertheless, the recurrence of this pattern across different therapeutic regimens suggests that successful treatment of CRLM requires more than generalized enhancement of T-cell activity.

In this review, a prognostic association denotes an outcome difference irrespective of treatment; predictive value requires evidence that liver status modifies the relative effect of an ICI-containing regimen, preferably through a treatment-by-liver-status interaction; and causal attribution requires perturbational or prospective mechanistic evidence. Most available clinical CRLM data establish association rather than validated prediction or direct causality. Accordingly, randomized comparative trials provide the strongest evidence for regimen efficacy, although unplanned liver-subgroup analyses remain exploratory; single-arm phase I/II trials estimate safety and activity but cannot isolate a liver-specific treatment effect; and retrospective cohorts remain hypothesis-generating because of treatment heterogeneity, selection bias and residual confounding.

### 3.1 Clinical evidence linking liver metastasis to immunotherapy resistance

The initial clinical signal emerged from the Japanese REGONIVO phase Ib study, in which regorafenib plus nivolumab produced an objective response rate of 33% and a median progression-free survival of 7.9 months in refractory MSS/pMMR mCRC. However, activity was substantially lower in patients with liver metastases, with an objective response rate of only 8.3%, whereas greater benefit was observed in patients with lung metastases without hepatic involvement (**Table 1**, Entry 1). Although the small sample size limited firm conclusions, this study raised the possibility that metastatic distribution could influence the efficacy of multikinase inhibitor–ICI combinations (38).

**Table 1:** Selected clinical evidence linking liver metastasis to immunotherapy resistance in microsatellite-stable colorectal cancer.

Entry	Treatment	Design and population	Overall activity	Liver-specific findings	Evidence level, inference and key limitation	Refs
1	Regorafenib plus nivolumab	Phase Ib; 24 patients with refractory MSS/pMMR mCRC	ORR, 33%; median PFS, 7.9 months	ORR was 8.3% in patients with liver metastases, whereas substantially greater activity was observed in patients with lung metastases without liver involvement	Phase Ib, single-arm; hypothesis-generating; small cohort, with no predictive or causal inference.	(38)
2	PD-1/PD-L1 inhibitors, including selected VEGFR inhibitor combinations	Retrospective cohort; 95 patients with refractory MSS mCRC receiving PD-1/PD-L1-directed therapy with or without investigational partners	Overall ORR, 8.4%	ORR was 0% in 54 patients with liver metastases versus 19.5% in 41 patients without liver metastases; median PFS was 1.5 versus 4.0 months, respectively	Retrospective, heterogeneous cohort; association only; residual confounding.	(10)
3	Regorafenib plus toripalimab	Phase Ib/II; refractory MSS mCRC	Clinical responses were observed in a selected subgroup	ORR was approximately 8.7% in patients with liver metastases versus 30.0% in those without liver metastases	Phase Ib/II, non-randomized; small exploratory subgroups; no comparator.	(39)
4	Regorafenib plus nivolumab	Multicenter, single-arm phase II; 70 patients with MSS/pMMR mCRC	ORR, 7.1%	No objective responses occurred in patients with liver metastases; ORR was 21.7% in patients without liver metastases	Prospective single-arm phase II; supports association, not an independent predictive effect.	(40)
5	Regorafenib, ipilimumab and nivolumab	Single-center, nonrandomized phase I; 39 patients with refractory MSS mCRC	At the recommended dose, ORR was 27.6%, median PFS was 4 months and median OS was 20 months	All objective responses occurred in patients without liver metastases; in this subgroup, ORR was 36.4% and median OS was not reached	Phase I, non-randomized; exploratory subgroup; no causal inference.	(41)
6	Botensilimab plus balstilimab	Expanded phase I; heavily pretreated MSS mCRC	In response-evaluable patients, ORR was 17% and DCR was 61%	ORR was 22% in patients without active liver metastases versus 0% in those with active liver metastases; median PFS was 4.1 versus 1.4 months, respectively	Expanded phase I; active-liver subgroup exploratory; no comparator.	(42)
7	PD-1/PD-L1 blockade with TKI or other investigational combinations	Retrospective analysis of MSS mCRC treated with different ICI-based regimens	Activity varied according to metastatic pattern	Patients with isolated lung metastases had better outcomes than patients with liver involvement; concurrent liver metastases reduced the apparent benefit associated with lung disease	Retrospective metastatic-pattern analysis; selection and disease-burden confounding.	(39)

A subsequent retrospective cohort of 95 patients receiving PD-1/PD-L1-directed therapy, with or without investigational partners, provided a larger, but still retrospective, association. No objective responses were observed among 54 patients with liver metastases, compared with an objective response rate of 19.5% among 41 patients without hepatic involvement. Median progression-free survival was also substantially shorter in the liver-metastatic group, at 1.5 versus 4.0 months (**Table 1**, Entry 2). Although treatment regimens were heterogeneous and the retrospective design precluded causal inference, the marked difference between the two groups indicated that liver involvement was associated with substantially reduced sensitivity to ICI-based therapy (10).

A phase Ib/II study of regorafenib plus toripalimab showed a similar pattern. The objective response rate was approximately 8.7% in patients with liver metastases, compared with 30.0% in those without hepatic disease (**Table 1**, Entry 3) (39). This result suggested that the negative effect of liver involvement was not confined to nivolumab-based treatment or to a single population. At the same time, the limited subgroup size and nonrandomized design mean that the magnitude of the difference should be interpreted cautiously.

The North American multicenter phase II study of regorafenib plus nivolumab further tested the reproducibility of the original REGONIVO findings. The overall objective response rate was only 7.1%, substantially lower than that reported in the Japanese study. Importantly, no objective responses occurred among patients with liver metastases, whereas the objective response rate reached 21.7% in patients without hepatic involvement (**Table 1**, Entry 4) (40). The contrast between Entries 1 and 4 suggests that the initial activity was not consistently reproduced and that vascular or stromal modulation alone may be insufficient in patients with established liver metastases; however, cross-study comparisons cannot establish a liver-specific treatment effect.

Adding CTLA-4 blockade was subsequently explored as a strategy to strengthen T-cell priming and effector function. In a nonrandomized phase I study, regorafenib, ipilimumab and nivolumab produced an objective response rate of 27.6% at the recommended dose, with a median progression-free survival of 4 months and median overall survival of 20 months. However, all objective responses occurred in patients without liver metastases; within that subgroup, the objective response rate was 36.4% and median overall survival had not been reached (**Table 1**, Entry 5) (41). Thus, activity was observed in selected patients, but the small, nonrandomized liver-metastasis subgroup does not establish whether CTLA-4 intensification can overcome hepatic resistance.

A similar limitation was observed with botensilimab plus balstilimab. Botensilimab is an Fc-enhanced anti-CTLA-4 antibody designed to improve T-cell priming and modulate suppressive immune populations, whereas balstilimab targets PD-1. In the expanded phase I cohort, the objective response rate was 17% and the disease control rate was 61% among response-evaluable patients. Nevertheless, the objective response rate was 22% in patients without active liver metastases and 0% in those with active hepatic disease; median progression-free survival was 4.1 and 1.4 months, respectively (**Table 1**, Entry 6) (42). This finding is particularly informative because it distinguishes active liver metastases from a previous history of treated hepatic disease, suggesting that ongoing liver tumor activity may be more relevant than liver involvement at any prior time.

Finally, an analysis based on metastatic distribution showed that patients with isolated lung metastases had better outcomes than those with liver involvement, whereas the presence of concurrent liver metastases reduced the apparent benefit associated with lung-dominant disease (**Table 1**, Entry 7) (39). This observation suggests that immunotherapy response may vary with the complete metastatic pattern rather than a binary liver-present/liver-absent variable. It is compatible with, but does not clinically prove, the hypothesis that hepatic lesions exert systemic immunoregulatory effects at other sites.

These examples show a notably consistent pattern: objective responses to mechanistically distinct ICI-based combinations are concentrated among patients without active liver metastases. However, most studies were early-phase, nonrandomized or retrospective, and definitions of active, treated or historical liver involvement were not uniform. Liver metastasis should therefore be considered a recurrent negative modifier of ICI efficacy rather than a fully validated predictive biomarker. Future studies should incorporate hepatic tumor volume, lesion number, previous liver-directed treatment and site-specific responses instead of relying only on a binary record of liver involvement. The consistency of this association across regimens strengthens its clinical relevance but does not, in the absence of prospective stratification or formal interaction testing, distinguish a predictive effect from adverse prognosis.

### 3.2 Antiangiogenic and multikinase inhibitor combinations

VEGF signaling connects abnormal vasculature with immune suppression and is therefore an attractive target in CRLM. Disorganized hepatic tumor vessels can limit lymphocyte trafficking, promote hypoxia and impair dendritic-cell maturation, while VEGF-associated signaling favors regulatory T cells and suppressive myeloid populations. Antiangiogenic agents may consequently enhance checkpoint blockade through vascular normalization, improved immune-cell entry and partial disruption of myeloid-mediated tolerance. Multikinase inhibitors can additionally affect stromal, oncogenic and macrophage-related pathways (43).

The clinical evidence summarized in **Table 1** nevertheless shows that this rationale is insufficient by itself. Regorafenib-based regimens produced early evidence of activity, but subsequent studies showed lower and less reproducible response rates, especially in patients with liver metastases (**Table 1**, Entries 1, 3 and 4). Differences in dose, patient population, prior treatment, microbiota and metastatic distribution may have contributed to these inconsistent results. A biologically plausible explanation is that regorafenib improves vascular access in selected tumors but does not fully correct macrophage-mediated T-cell deletion, deficient antigen presentation or dense hepatic stromal barriers.

Other antiangiogenic combinations have yielded similarly mixed results. In the randomized BACCI study, adding atezolizumab to capecitabine and bevacizumab produced only a modest improvement in progression-free survival in refractory mCRC. Exploratory analyses suggested greater activity in patients without liver metastases, whereas patients with hepatic disease obtained little incremental benefit from PD-L1 blockade. Although this study was not powered to validate a liver-specific predictive effect, its findings were consistent with the regorafenib-based evidence (44).

Lenvatinib plus pembrolizumab initially showed encouraging activity in a nonrandomized CRC cohort, but the randomized phase III LEAP-017 trial did not demonstrate a statistically significant improvement in overall survival over standard late-line therapy in pMMR or non-MSI-H mCRC (45, 46). Similarly, the phase III IMblaze370 trial found that atezolizumab with or without cobimetinib did not improve overall survival compared with regorafenib in predominantly MSS mCRC (47). These negative trials illustrate the limitations of advancing broad antiangiogenic or targeted therapy combinations on the basis of early activity without adequately resolving organ-specific resistance.

Fruquintinib and other VEGFR-directed agents are also being combined with PD-1 antibodies. Small prospective and real-world studies have reported manageable activity, but randomized validation is lacking, and benefit again appears enriched in patients without liver metastases (48, 49). Antiangiogenic combinations therefore remain biologically relevant, but their efficacy probably depends on whether vascular exclusion is the dominant resistance mechanism. Lesions characterized mainly by myeloid deletion, defective antigen presentation or extensive fibrosis may require additional or alternative intervention.

### 3.3 Targeting myeloid, stromal and innate immune resistance

The limitations of checkpoint and antiangiogenic combinations provide a rationale for targeting the cellular systems that maintain hepatic tolerance. Myeloid-directed approaches include inhibition of CSF1–CSF1R, CCL2–CCR2, CCR5, CXCR2 or PI3Kγ signaling, together with modulation of TREM2, CD47–SIRPα and CD40. These strategies seek to reduce recruitment of suppressive monocytes and neutrophils, reprogram macrophages or improve phagocytosis and antigen presentation. CCR2 or CSF1R blockade may restrict monocyte-derived macrophage accumulation, whereas CXCR2 inhibition may reduce suppressive neutrophil trafficking. CD40 agonists differ conceptually because they aim to activate antigen-presenting cells rather than simply deplete myeloid populations (50, 51).

The supporting evidence is uneven and should not be treated as a single class. CCR2 has relatively direct CRLM evidence from human metastatic specimens and murine mechanistic studies, but not from a CRLM-specific randomized trial (52). CXCR2 is supported by human CRLM profiling and immunocompetent or spontaneous-metastasis models linking neutrophil recruitment to hepatic metastatic growth, whereas clinical reversal of ICI resistance remains unproven (53). TREM2 evidence is emerging from CRLM multi-omic profiling and preclinical functional experiments (54). By contrast, CSF1R and PI3Kγ are supported predominantly by non-CRLM tumor models and early solid-tumor development, and CD47–SIRPα evidence in CRC is mainly preclinical and is not liver-metastasis-specific (55). None of these targets is currently a validated CRLM-specific therapeutic biomarker; all should be presented as investigational.

Despite a strong mechanistic rationale, clinical evidence specifically demonstrating reversal of CRLM-associated ICI resistance remains limited. Most agents have been evaluated in early-phase solid-tumor studies without liver-specific response analyses or paired CRLM biopsies. Moreover, indiscriminate myeloid depletion could remove macrophage or dendritic-cell populations that support phagocytosis, antigen presentation or tertiary lymphoid structure formation. Selective reprogramming of suppressive myeloid states may therefore be more rational than broad elimination of the entire hepatic myeloid compartment.

Stromal intervention represents a second strategy. TGF-β signaling promotes CAF activation, extracellular-matrix deposition, immune exclusion and suppressive myeloid differentiation. TGF-β inhibitors, bifunctional PD-L1/TGF-β agents, CXCL12–CXCR4 inhibitors and FAP-directed approaches have therefore been developed to improve lymphocyte access. However, clinical translation has been difficult because TGF-β has context-dependent physiological functions, systemic inhibition can cause toxicity and fibroblast populations are functionally heterogeneous. Some stromal states support lymphocyte organization and tertiary lymphoid structure formation. Accordingly, stromal normalization focused on the fibrotic and chemokine programs responsible for T-cell exclusion may be preferable to nonselective CAF depletion (56, 57).

Restoring innate immune sensing may address an earlier defect in the cancer–immunity cycle. STING and Toll-like receptor agonists, CD40 agonists, tumor-directed IL-12, oncolytic viruses and cancer vaccines can promote dendritic-cell activation, inflammatory cytokine production and *de novo* T-cell priming. These approaches may be particularly relevant when hepatic lesions contain few antigen-presenting cells or pre-existing tumor-reactive lymphocytes. Unlike additional checkpoint blockade, which principally acts on an existing immune response, innate immune agonists seek to generate or rebuild that response. Systemic toxicity, rapid hepatic clearance and limited intratumoral delivery remain major challenges, making locoregional administration or tumor-targeted formulations potentially important in CRLM (58).

Antiangiogenic–ICI combinations have the most extensive clinical experience but inconsistent efficacy in active liver metastases. Myeloid- and stromal-directed therapies have strong biological foundations but remain incompletely validated clinically, whereas innate immune activation offers a strategy to restore priming but faces delivery and safety barriers. These approaches are not mutually exclusive, and selected patients may require coordinated intervention at several levels.

### 3.4 Why current combinations frequently fail

The recurring failure of biologically plausible combinations indicates that MSS CRC, particularly CRLM, cannot be sensitized through nonspecific treatment intensification. Several explanations emerge from the clinical studies summarized in **Table 1**. First, many regimens intensify T-cell activation without restoring the upstream processes required to generate effective tumor-reactive lymphocytes. PD-1 and CTLA-4 blockade cannot compensate for severe dendritic-cell deficiency, poor antigen presentation or deletion of tumor-reactive clones. This may explain why stronger checkpoint combinations remained largely ineffective in active liver metastases (**Table 1**, Entries 5 and 6).

Second, vascular normalization may improve immune-cell access without removing suppressive macrophages, stromal barriers or metabolic stress. This limitation is evident in the inconsistent activity of regorafenib-based combinations (**Table 1**, Entries 1, 3 and 4). Antiangiogenic intervention may benefit tumors dominated by vascular exclusion but is unlikely to reverse all layers of hepatic tolerance.

Third, clinical trials frequently treat MSS mCRC as a single disease entity. Patients are selected by MMR status without prospective stratification by metastatic organ, liver tumor burden, active versus treated hepatic disease, stromal phenotype or myeloid composition. A regimen with meaningful activity in a biologically defined subgroup may therefore appear ineffective in an unselected population, whereas an early single-arm signal may be inflated by enrichment for patients without liver metastases.

Fourth, preclinical models often fail to reproduce the relevant resistance state. Highly immunogenic subcutaneous CT26 or MC38 tumors may respond to strategies that fail when the same cells are established orthotopically or within the liver. The clinical failure of atezolizumab plus cobimetinib after encouraging preclinical data illustrates this model-to-patient disconnect.

These findings support a mechanism-matched approach. Patients with vascular exclusion may benefit from angiogenic normalization, those with dominant myeloid suppression may require macrophage or neutrophil reprogramming, and those with defective priming may require innate immune activation or vaccination. Fibrotic lesions may need stromal intervention, whereas high-volume hepatic disease may require local reduction before systemic immunity can be restored. The therapeutic objective is therefore not simply to identify a stronger combination, but to determine which immune barrier dominates each CRLM and intervene at the appropriate stage of the cancer–immunity cycle. Operationally, a priming-deficient lesion would justify a priming-restoration strategy; a myeloid-deletion phenotype, selective myeloid reprogramming; a vascular or stromal-exclusion phenotype, vascular or matrix normalization; and high-volume active hepatic disease, comprehensive local control combined with correction of residual systemic resistance. Evidence maturity is a separate gate: combinations supported only by CRLM models should enter biomarker-rich prospective trials rather than be described as established clinical options.

## 4 Integrating liver-directed and systemic immunotherapy

Colorectal cancer liver metastases (CRLMs) may act not only as sites of tumor burden but also as reservoirs of local and systemic immune suppression. Liver-directed treatments, including resection, thermal ablation, radiotherapy and hepatic arterial therapy, can reduce hepatic disease, release tumor antigens and alter the surrounding stromal and myeloid environment. These effects provide a rationale for combining local interventions with systemic immunotherapy. However, local tumor destruction does not consistently restore antitumor immunity, and its biological consequences depend on treatment completeness, modality, disease distribution and sequencing.

The broader HCC literature provides useful hypotheses regarding ICI–antiangiogenic combinations, regional antigen release and transarterial chemoembolization (TACE) (59), but it is not efficacy evidence for CRLM. HCC is a primary hepatic malignancy that commonly develops in chronically inflamed or cirrhotic liver and has vascular biology and treatment standards distinct from metastatic colorectal adenocarcinoma in an often noncirrhotic liver. Accordingly, HCC response estimates are not extrapolated here; TACE is not presented as a validated CRLM strategy, and the CRLM claims below are based on disease-specific studies of resection, ablation, stereotactic radiotherapy, Y-90 radioembolization or hepatic arterial infusion.

### 4.1 Local control and immune modulation

Resection or complete ablation may reduce suppressive cytokine production, myeloid recruitment and sequestration of tumor-reactive T cells. Thermal ablation can additionally release tumor antigens and damage-associated signals, potentially promoting dendritic-cell activation and T-cell priming (60). Nevertheless, these effects are often incomplete. Antigen release may occur without effective immune priming, while residual hepatic tissue remains enriched in tolerogenic macrophages and regulatory signals. Incomplete ablation may generate a hypoxic periablational zone that promotes accelerated outgrowth of residual metastatic cells, thereby compromising local control (61).

Clinical findings support this caution. Partial liver-directed ablation combined with durvalumab and tremelimumab failed to induce responses in untreated hepatic lesions, suggesting that local antigen release alone is insufficient to overcome the broader immunosuppressive liver environment (62). More complete hepatic control, together with strategies that restore antigen presentation or reduce myeloid suppression, may therefore be required.

Radiotherapy and radioembolization provide related opportunities. SBRT can induce DNA damage, antigen release and cGAS–STING signaling, whereas Y-90 radioembolization enables liver-selective radiation delivery (63). However, radiation may also deplete lymphocytes and induce TGF-β, hypoxia and myeloid recruitment. Evidence that either modality consistently converts MSS CRLM into an ICI-responsive state remains preliminary (64). Hepatic arterial infusion chemotherapy may offer a complementary strategy by delivering high local drug concentrations while permitting concurrent systemic therapy. Studies combining HAI with systemic chemotherapy have reported high hepatic response and conversion-to-resection rates, but evidence supporting the additional use of immune-checkpoint blockade in MSS CRLM remains limited (65). Regional delivery of immune therapeutics, including CEA-directed CAR-T cells, is also feasible but remains investigational (66).

### 4.2 Sequencing and patient selection

The optimal sequence of local and systemic therapy remains unresolved. Local treatment before immunotherapy may reduce hepatic immune suppression, whereas preoperative or pre-ablation immunotherapy may expand tumor-reactive T-cell clones while antigen remains available. Concurrent treatment could increase biological interaction but may also heighten hepatic toxicity or expose activated lymphocytes to radiation and perioperative immunosuppression.

Local–systemic integration is most plausible in liver-limited or oligometastatic disease where dominant lesions can be comprehensively treated (67). In extensive bilobar disease or uncontrolled extrahepatic progression, partial treatment of hepatic lesions is unlikely to reverse the total immunosuppressive burden. Future trials should therefore consider liver tumor volume, number and distribution of metastases, completeness of local treatment, baseline immune features and the presence of extrahepatic disease. Responses should also be reported separately for treated liver lesions, untreated hepatic lesions and extrahepatic sites. At minimum, hepatic disease should be classified as (i) active—untreated or radiographically progressive measurable disease; (ii) residual—known or suspected viable tumor after local or systemic therapy; (iii) previously treated—historical hepatic lesions without a contemporaneous assessment of viability; or (iv) completely controlled—all known hepatic lesions resected, ablated or in durable complete radiographic response. Time since treatment and the method used to establish control should also be recorded, because these states are unlikely to exert equivalent immunological effects.

Liver-directed therapy should not be considered a generic add-on to checkpoint blockade. Its immunological value depends on whether it meaningfully reduces the hepatic suppressive compartment and whether systemic therapy simultaneously addresses residual defects in antigen presentation, myeloid regulation or T-cell function.

## 5 Biomarkers, models and future directions

Biomarker claims should be separated by intended use and validation level. MMR/MSI status is a clinically validated predictive biomarker for ICI selection in CRC, but no liver-specific biomarker has been validated to predict incremental benefit from an ICI-containing regimen within MSS CRLM. CD8^+^ T-cell density and localization at the invasive margin have prognostic associations in CRLM, including association with chemotherapy response, but are not validated predictors of ICI benefit (68). Postoperative ctDNA has prospective prognostic and recurrence-monitoring value after CRLM resection, yet it is likewise not an established ICI-predictive marker (69). Liver-metastasis status is a recurrent negative clinical modifier, not a validated stand-alone predictive biomarker. Myeloid-cell states, CAF/TGF-β activity, vascular or hypoxic signatures and tertiary lymphoid structures remain exploratory candidates. Changes in paired biopsies, circulating immune cells or ctDNA should be considered pharmacodynamic or response-monitoring readouts until prospective studies demonstrate treatment-specific predictive value.

No single model reproduces the complete hepatic immune microenvironment, so model selection should follow the biological question. Orthotopic cecal models preserve primary-site interactions and portal dissemination but produce liver metastases with variable frequency and latency. Spontaneous-metastasis models retain invasion, circulation, hepatic seeding and immune evolution, although they are slower, heterogeneous and difficult to standardize. Portal, intrasplenic or intrahepatic implantation gives reproducible hepatic burden and exposes tumors to Kupffer cells, liver sinusoidal endothelial cells and stellate cells, but bypasses part or all of the metastatic cascade and may introduce procedure-related inflammation. Syngeneic organoid transplantation preserves engineered or patient-relevant tumor genotypes and architecture while permitting an intact mouse immune system, yet its stromal and myeloid compartments are reconstructed after transplantation (70). Humanized patient-derived xenograft or organoid systems allow testing of human targets but incompletely reconstitute Kupffer cells, liver sinusoidal endothelium, myeloid diversity and HLA-matched immunity, and may be limited by graft-versus-host effects. Robust translation therefore requires complementary models and confirmation that the selected system reproduces the relevant hepatic resident-cell, spatial and immune-suppressive features.

Prospective immunotherapy trials should stratify patients by active, residual, previously treated and completely controlled liver disease rather than by liver-metastasis presence or absence alone. Hepatic tumor volume, number and distribution of lesions, time since and completeness of local treatment, and extrahepatic disease should be recorded. Responses should be reported separately for treated liver lesions, untreated hepatic lesions and extrahepatic sites, and prespecified treatment-by-hepatic-state interaction analyses are needed before claiming predictive value. Mechanistic endpoints should accompany objective response, progression-free survival and overall survival so that clinical association can be linked, where possible, to biological change.

Ultimately, CRLM should be regarded not merely as an anatomical site of disease but as a distinct immunological compartment. Effective treatment is therefore unlikely to result from indiscriminate intensification of checkpoint blockade. Instead, future strategies should combine appropriate hepatic disease control with interventions matched to the dominant resistance mechanism, including defective immune priming, myeloid suppression, stromal exclusion or vascular–metabolic dysfunction. Such an organ-informed approach may provide a more rational path toward extending immunotherapy benefit to patients with MSS colorectal cancer and active liver metastases. The resulting state–mechanism–evidence framework is the principal conceptual advance of this review: it distinguishes the activity and control status of hepatic disease, identifies the dominant immune bottleneck, and limits therapeutic recommendations according to the maturity and CRLM specificity of the evidence. This differs from broader HCC combination frameworks, which are valuable for hypothesis generation but cannot substitute for CRLM-specific validation.
